# Effect of Degradation During Multiple Primary Mechanical Recycling Processes on the Physical Properties and Biodegradation of Commercial PLA-Based Water Bottles

**DOI:** 10.3390/polym17182542

**Published:** 2025-09-20

**Authors:** Cristina Muñoz-Shugulí, Diana Morán, Eliezer Velásquez, José Manuel López-Vilariño, Carol López-de-Dicastillo

**Affiliations:** 1 Faculty of Sciences, Escuela Superior Politécnica de Chimborazo (ESPOCH), Riobamba 060106, Ecuador; cristina.munoz@espoch.edu.ec; 2 Group for Research and Innovation in Food Packaging, Riobamba 060107, Ecuador; 3 Packaging Group, Institute of Agrochemistry and Food Technology (IATA-CSIC), Av. Agustín Escardino 7, Paterna, 46980 Valencia, Spain; dmoran@iata.csic.es; 4 Packaging Innovation Center (LABEN-Chile), University of Santiago of Chile (USACH), Santiago 9170201, Chile; eliezer.velasquez@usach.cl; 5 Hijos de Rivera SAU, José María Rivera Corral 6, 15008 A Coruña, Spain; jmlopez@estrellagalicia.es

**Keywords:** recycling, polylactic acid, biopolymer, food packaging, biodegradation

## Abstract

For sustainable development aligned with circular economy principles, the recycling of biopolymers such as polylactic acid (PLA) is of growing interest. In this study, the effect of primary recycling through repeated mechanical reprocessing was investigated. PLA water bottle preforms were subjected to six consecutive extrusion cycles, and changes in its molecular structure and physical properties were evaluated. Structural analysis revealed a progressive degradation, evidenced by a great reduction in the molar mass and increase in the melt flow index, attributed both to the chain scission derived from the thermal degradation and shear stresses of the extrusion process, and hydrolysis at the ester linkage of the polymer. Recycled samples exhibited a darkening of the color and a continuous decrease in thermal stability. After six reprocessing cycles, PLA crystallinity increased from 6.9 to 39.5%, the cold crystallization process disappeared, and molecular weight reduced by up to 40%. Barrier properties were highly affected after reprocessing and by the increase in relative humidity. Biodegradation tests revealed that crystallinity affected considerably the biodegradation rate of PLA. Although the molecular weight was considerably reduced during reprocessing, the biodegradation was slowed down. These findings provide insights into the limitations and potential of mechanically recycled PLA for future material applications.

## 1. Introduction

The growing global demand for plastic materials has led to an unprecedented rise in plastic waste, significantly contributing to environmental pollution and resource depletion. Among the most used biopolymers, poly(lactic acid) (PLA) has garnered considerable attention as a biodegradable and bio-based alternative to traditional petroleum-derived plastics. PLA is primarily derived from renewable resources such as corn or sugarcane, making it a sustainable option for applications such as food packaging, disposable cutlery, and water bottles [1]. In addition, PLA is known as a compostable polymer which is considered a great solution to avoid plastic residue accumulation; however, specific conditions could be necessary to break it down [2]. Therefore, despite its environmental advantages over conventional plastics, the end-of-life management of PLA still represents a challenge.

While PLA is biodegradable under specific industrial composting conditions, its recycling process remains underdeveloped, largely due to its distinct properties compared to other commonly used plastics, such as polyethylene terephthalate (PET). The challenge in recycling PLA stems from its relatively high crystallinity and the thermal degradation that occurs during conventional recycling methods [3]. As a result, much of PLA waste ends up in landfills or incinerators, undermining its potential as a sustainable material [4]. This issue highlights the urgent need for effective reprocessing technologies that can extend the lifecycle of PLA products, reduce waste, and recover valuable resources.

Mechanical recycling is considered the end-of-life option with the lowest environmental impact because of the ability to recover valuable resources [5]. The importance of recycling cannot be overstated, particularly in the context of addressing the ongoing plastic waste crisis. Recycling PLA not only helps mitigate the environmental impact associated with landfill accumulation and incineration but also reduces energy consumption and greenhouse gas emissions, and conserves valuable resources by enabling their use on the production of new products. By reprocessing PLA, manufacturers can reduce their dependence on fossil fuel-derived plastics and create a circular economy where materials are continually recycled and reused as an alternative to PLA composting, which depends on the availability of industrial composting plants and specific conditions of temperature and humidity [6]. Life cycle assessment (LCA) studies consistently show that key impact categories such as climate change, human toxicity, and fossil resource depletion are all minimized with mechanical recycling, primarily due to lower energy requirements and the avoidance of virgin material production [7,8]. Maga et al. (2019) reported that substituting virgin PLA with recyclates, while considering factors such as market price and molecular weight loss, could provide a credit of 1070 kg CO_2_ eq per functional unit, with the mechanical recycling of 1 t of post-consumer PLA potentially avoiding up to 793 kg CO_2_ eq of emissions [9].

However, for this to be a feasible and sustainable option, significant advancements are required in understanding the properties of the material after mechanical recycling, encompassing both primary and secondary reprocessing. Primary mechanical recycling is aimed at reprocessing materials that have not reached their final use, and the recycling product is used for the same purpose as virgin polymers [10]. On the contrary, secondary mechanical recycling consists of the reprocessing of materials that have been previously used, and therefore, their cleaning is necessary [11]. Several studies have focused on the analysis of mechanical, thermal, and optical properties of reprocessed and recycled pristine PLA after multiple recycling cycles [5,12]. For instance, Shojaeiarani et al. (2019) highlighted the reduction in storage modulus and molecular weight after five reprocessing cycles of pristine PLA [13]. Finnerty et al. (2023) showed the discoloration and decrease in tensile properties of natural-fiber-reinforced PLA, in addition to the increase in glass transition temperature and crystallinity of PLA after six recycling processes [14]. Likewise, Beltrán et al. (2021) reported that recycling 3D-printed PLA waste triggers the increase in the crystallinity and a decrease in the degradation time of the polymer by the presence of shorter chains [15]. It is critical to determine the impact of reprocessing on PLA’s performance to ensure that recycled materials meet the required standards for new products.

However, the plastics used in food and beverage packaging are not exclusively composed of polymers but usually contain a number of additives that can affect polymer degradation and recyclability. These additives include stabilizers (e.g., antioxidants, UV absorbers, and hindered amine light stabilizers) used to inhibit thermal and photo-oxidative degradation, such as plasticizers, fillers (e.g., calcium carbonate, talc), and pigments that modify rheology, crystallization, or color [15,16].

In this context, this work explores the potential for reprocessing commercial PLA-based water bottles, focusing on the evaluation of reprocessed plastics in terms of their structural integrity and thermal, processing, and barrier properties for use in new product applications, but also the effect of reprocessing on the biodegradation rate and molar mass distribution. For that, real commercial PLA-based water bottle preforms were subjected to six consecutive melt extrusion cycles, a processing route that closely mimics industrial plastic reprocessing practices. Throughout these cycles, the structural, thermal, and barrier properties of the material were systematically monitored in order to assess the influence of the mechanical and thermal stresses imposed by extrusion. This approach allows us to evaluate, under realistic conditions, the feasibility of employing recycled PLA materials in the production of new plastic products.

The novelty of this work lies in its industrial relevance and comprehensiveness. Unlike previous studies that have often relied on pure PLA grades and laboratory-scale processing methods, our investigation focuses on commercial PLA formulations designed for packaging applications, which inherently include additives required for industrial processing. By combining multiple reprocessing cycles with the assessment of biodegradation behavior and molar mass distribution, this study provides a more realistic and holistic understanding of the performance of PLA under primary recycling conditions, offering insights that are directly translatable to real-world applications.

## 2. Materials and Methods

### 2.1. Chemicals, Reagents, and PLA Commercial Water Bottle Preforms

Commercial green PLA-based water bottle preforms were supplied by Cabreiroá (Hijos de Rivera S.A., A Coruña, Spain). Ultrapure water was obtained from a Milli-Q Plus purification system (Millipore, Molsheim, France). Silica gel 2.5–6 mm with an indicator (without cobalt chloride) was purchased from PanReac (Barcelona, Spain).

### 2.2. Reprocessing Cycles and Samples: PLA0 to rPLA6

**Figure 1:**
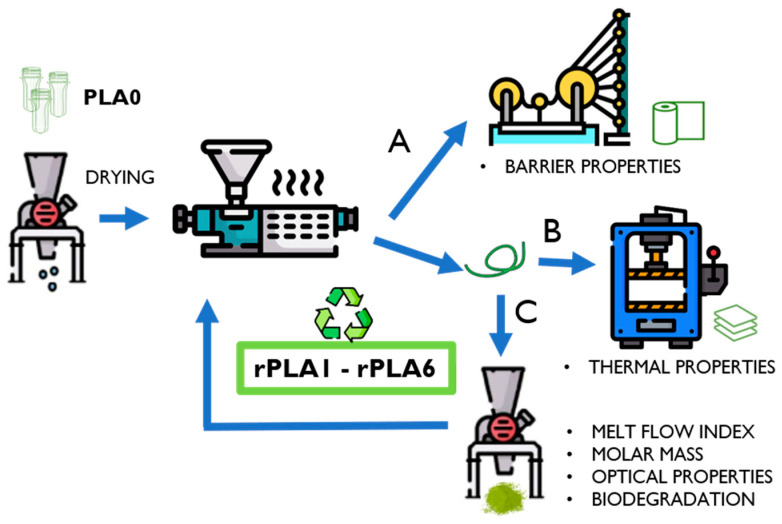
Description of the methodology and characterization of reprocessed PLA-based water bottles: (A) cast extrusion; (B) compression molding; and (C) grinding.

Commercial PLA water bottle preforms were subjected to consecutive reprocessing cycles in order to understand the effect of multiple primary recycling process on PLA-based water plastic bottles. Figure 1 summarizes a design of the methodology including three types of transformation processes that were carried out in order to analyze specific properties. Plastic water preforms based on PLA (PLA0) were ground by using a rotating steel blade in a SM 300 cutting mill (Retsch, Asturias, Spain) at 1800 rpm with a 4 mm diameter sieve. Subsequently, PLA0 was dried under vacuum at 40 °C overnight and then processed through melt extrusion in a co-rotating twin-screw mini-extruder (MC 15 HT, L/D = 30) (Xplore, Barcelona, Spain) at 180 °C, 100 rpm, and a residence time of 3 min, obtaining the first reprocessed rPLA1 material. The filament obtained from the extrusion was ground, dried, and extruded under the same previously described conditions, and this process was carried out six times (extrusion cycles) for obtaining rPLA1, rPLA2, rPLA3, rPLA4, rPLA5, and rPLA6 samples.

Depending on the property to be characterized, the reprocessed rPLA samples were either analyzed after grinding or were subjected to further processing and transformation into films. On the one hand, the ground reprocessed rPLA samples were analyzed by optical, molar mass, and melt flow index assays. Meanwhile, the structural and thermal characterization was performed by Fourier transform infrared spectroscopy, differential scanning calorimetry, and thermogravimetry, respectively, by using thermo-compressed rPLA films. For that, the ground material was processed through compression molding by using a Micro Scientific LP20-B press (Labtech Engineering, Samut Prakan, Thailand) at 180 °C, 39 bar, and 2 min of cooling to obtain films with an approximate thickness of 100 ± 20 µm. On the other hand, casted films with high homogeneity in thickness were obtained for analyzing the barrier properties by using a roll-to-roll system coupled to the extruder (see the display in Figure S1 in Supplementary File). Rolls were heated at 210 °C and set at 800, 848, and 44 mm min^−1^ in the extruded melt to winding roll direction. Figure S1 includes pictures of corresponding equipment.

### 2.3. Characterization of Reprocessed PLA-Based Water Bottles

#### 2.3.1. Structural Characterization

The Fourier transform infrared (FTIR) spectra of the samples were analyzed in order to observe any structural changes in PLA during reprocessing. Spectra were measured between 4000 and 600 cm^−^^1^ with 64 scans and a resolution of 4 cm^−^^1^ using a Jasco 4100 FTIR spectrometer (Jasco, Easton, MD, USA) with a single reflection attenuated total reflectance (ATR) accessory (ZnSe crystal, PIKE Technologies, Madison, WI, USA). The FTIR-ATR spectra were corrected using Spectra Manager version 2.15.01 and normalized with the band at 1453 cm^−^^1^ as the internal standard of PLA [17].

#### 2.3.2. Molar Mass Distribution (MMD)

The number average molar mass ($\bar{M_{n}}$), weight average molar mass ($\bar{M_{w}}$), and dispersity Ð ($\bar{M_{w}}/\bar{M_{n}}$) of samples were measured by gel permeation chromatography (GPC) using a Separation Module-Alliance 2695 chromatograph (Waters, Barcelona, Spain) coupled to an ultraviolet (UV) detector at 220 nm and Waters Styragel HR 5E, 4E, and 3 HPLC columns combined in series. The mobile phase employed was tetrahydrofuran (THF) with a flow rate of 0.3 mL min^−^^1^ at 35 °C and an injection volume of 10 µL. Calculation of the molar mass distribution was carried out relative to certified mass polystyrene standards (580–3,080,000 Da).

#### 2.3.3. Melt Flow Index (MFI) Analysis

The measurement of the melt flow index of the samples was carried out following ISO 1133-1:2012 [18] with CEAST MF20 MFI equipment (Instron, Barcelona, Spain). The tests were carried out with a 2 mm diameter nozzle at a temperature of 190 °C and a mass of 2.16 kg. The elapsed time between the two measurements was 5 s.

#### 2.3.4. Optical Characterization

The change in color derived from the primary recycling process of PLA bottle preforms was evaluated following the ASTM E313 method in a CM-3500 colorimeter (Konica Minolta, Dietikon, Switzerland) with SpectraMagic NX software (ver. 3.40). The instrument was calibrated using a blank, and the samples were measured with a standard cylindrical accessory for black (no light reflection) with a D65 illuminant and a standard observer of 10°. The measurements were taken in triplicate, and results were expressed on the CIELab color scale by determining L*, a*, and b* parameters that showed lightness, reddish/greenish, and yellowish/bluish, respectively. The color difference (ΔE*) was calculated with respect to the original bottle preform (PLA0), which was chosen as the reference sample, and using Equation (1):

(1) $$∆E*=\sqrt{({∆L*)}^{2}+({∆a*)}^{2}+{\left(∆b*\right)}^{2}}$$

The color evaluation was carried out according to Navneet et al.’s (2024) description, where a ΔE* < 1 is imperceptible, a ΔE* value between 1 and 2 shows that only an experienced observer notices the difference, a ΔE* value between 2 and 3.5 expresses a notable difference for an inexperienced observer, a ΔE* value between 3.5 and 5 confirms a clear and perceptible difference, and a ΔE* ≥ 5 represents a different color for the observer [19].

#### 2.3.5. Thermal Properties

Thermogravimetric analysis (TGA) was performed with approximately 10 mg of sample by using TGA 550 equipment (TA Instruments, New Castle, DE, USA). Samples were heated in alumina crucibles (70 µL) from 30 to 800 °C at 20 °C/min under a nitrogen atmosphere. The T_deg_ was determined by the maximum mass loss rate value. Thermal transitions were determined by differential scanning calorimetry (DSC) by using a DSC Q2000 (TA Instruments, New Castle, DE, USA). Approximately 10 mg of samples was placed in sealed aluminum pans and submitted to a thermal program consisting of two heating and one intermediate cooling cycles. Heating was set up from −20 °C to 220 °C while cooling from 220 °C to −10 °C. All cycles were carried out at 10 °C min^−1^ under nitrogen flow with an isotherm of 2 min between them. The glass transition (T_g_), cold crystallization temperature (T_cc_), melting temperature (T_m_), and their corresponding enthalpies (ΔH_cc_ and ΔH_m_) were determined from the second heating by TA Instruments Universal Analysis 2000 software. The second heating process allowed studying the effect of the molar mass of successively reprocessed PLA under controlled crystallization without considering the thermal history. Crystallinity fraction (χ_c_) was calculated from the values obtained in the second heating:

(2) $$\chi_{c}\left(\%\right)=\frac{{∆H}_{m}-{∆H}_{cc}}{{∆H}_{m}^{0}}\times 100$$

where the ${∆H}_{m}^{0}\;$ corresponds to 93.1 J g^−1^ as the theoretical melting enthalpy of 100% crystalline PLA [20].

#### 2.3.6. Barrier Properties

The oxygen (OP) and water vapor permeability (WVP) of samples were determined at 50% and 90% of relative humidity (RH) and 23 °C. The OP was determined in an OXTRAN 2/21ML Mocon (Lippke, Neuwied, Germany), where a cell conditioning process of 6 h was programmed, and the oxygen transmission rate was registered every 45 min until a constant value was reached. Water vapor permeability (WVP) assay was carried out following ISO 2528:2017 by using Payne permeability cups (Elcometer, Manchester, UK) with a permeation surface of 4.91 cm^2^ [21]. To ensure the necessary relative humidities, the cups were then stored in desiccators containing Mg(NO_3_)_2_, 6H_2_O, and BaCl_2_·2H_2_O salt solutions for 50 and 90% RH, respectively. The cups were weighed daily, and the plot of the weight increment versus time provided the water vapor transmission rate. These values were then divided by the water pressure gradient and multiplied by the sample thickness to obtain the WVP value. WVP results are displayed as the average of at least three samples in kg m m^−2^ s^−1^ Pa ^−1^

### 2.4. Biodegradation Studies

The effect of primary recycling on biodegradation was evaluated by analyzing the determination of the biodegradability of reprocessed PLA0 and reprocessed rPLA1 and rPLA6 under controlled aerobic composting conditions according to UNE-EN ISO 14855-2:2018 [22]. This method is based on the measurement of carbon dioxide generated by gravimetry [23,24].

Stabilized mature compost was used as the inoculum. The test material was mixed with the inoculum in a 1:6 ratio (dry weight) and sand (320 g in dry weight, adding water to obtain a moisture content of 15%) introduced into a 3 L static vessel where biodegradability was performed under optimal aerobic conditions of temperature and humidity for a test period of four months. The test was carried out at a constant temperature of 58 ± 2 °C. Air was supplied inside the reactors to ensure aerobic conditions throughout the test. In addition, the test vessels were shaken twice a week to ensure homogeneous air distribution throughout the vessel. The amount of carbon dioxide generated was absorbed in a column with sodium lime and measured at time intervals by weighing the grams of CO_2_ retained by means of a precision electronic balance. The percentage of biodegradation was calculated by the ratio between the carbon dioxide generated from the test material and the theoretical maximum amount of carbon dioxide (ThCO_2_) that can be produced from the test material. A blank sample (compost without sample) and a reference control sample based on cellulose were also analyzed. The analyses were conducted in triplicate bioreactors for each sample, and the average and standard error values are reported. The supporting tests were the determination of total Nitrogen (Nt) and Total Organic Carbon (TOC).

### 2.5. Statistical Analysis

All data were expressed as mean ± standard deviation (SD). Analysis of variance (ANOVA) was carried out for comparisons, using InfoStat software, version 2020 (Cordoba, Argentina) with a significance value of α = 0.05, followed by an LSD test to determine differences between mean values when necessary.

## 3. Results

### 3.1. Structural and Optical Properties After Multiple Mechanical Recycling

**Figure 2:**
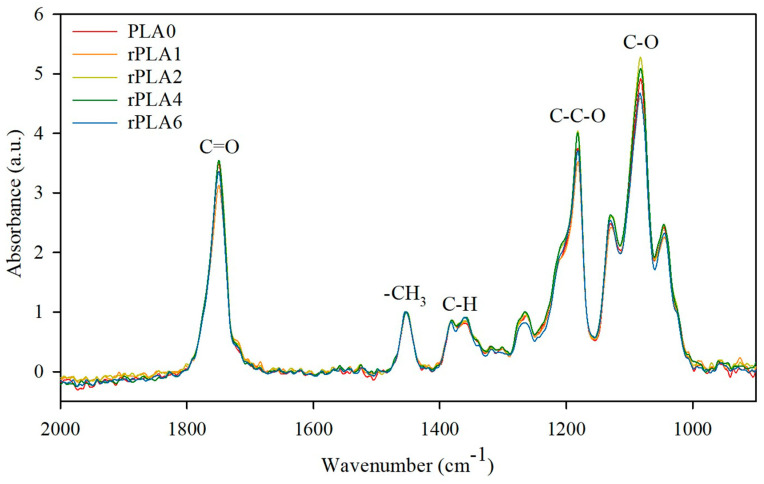
FTIR spectra of reprocessed PLA.

Figure 2 shows the FTIR spectra of PLA0 and subsequent reprocessed materials. Characteristic FTIR bands of PLA were observed for all samples at 1755 cm^−1^, attributed to the C=O stretching, between 1300 cm^−1^ and 1500 cm^−1^ related to the symmetric and asymmetric deformational bending vibrations of C–H from the –CH_3_ group, and between 1000 cm^−1^ and 1200 cm^−1^ assigned to the C–O and C–C–O bonds [25]. However, a slight change in band width at 1755 cm^−1^ was observed, without wavenumber displacement. Thus, the band was thinned when PLA0 was reprocessed, and it recovered the width by the following reprocessing cycles. The initial change from PLA0 to rPLA1 could be attributed to the degradation through the common interaction of hydrogen bonds that results in the formation of lactic acid and alcohol groups [26,27]. However, reactions of combination between species of chains with hydroxyl ends, and between species with esterified ends generated by esterification, could take place at the same time, and thus, changes in the FTIR spectrum of reprocessed PLA were not observed. This is in accordance with the reaction degradation mechanism proposed for PLA by Velásquez et al. (2021) [28].

Monitoring the molar mass of PLA-based bottles over the reprocessing is important since one of the main degradation processes of polyester chains is their chain scission [29]. Table 1 shows the progressive reduction of the molar mass ($\bar{M_{w}}$ and $\bar{M_{n}}$) after reprocessing cycles. The greatest drop in molar mass occurred after the first reprocessing cycle, achieving around 16% reduction with respect to PLA0. Subsequently, this reduction was less between processing, with a decrease in molar mass between 4 and 7% from the previous value occurring after each reprocessing.

$M_{w}$ reduced by up to 40% after six reprocessing cycles (rPLA6) (see Figure S2 in Supplementary Material). These values were within the range of molar mass reduction (34 to 40%) after five instances of reprocessing PLA filaments for 3D printing [30,31]. The reduction in the molar mass was attributed to the chain scission derived from the thermal degradation, shear stresses of the extrusion process, and hydrolysis at the ester linkage of the polymer [27]. Nevertheless, Table 1 shows that the reduction in the molar mass occurred in number and in weight, so that the dispersity was not modified, being 2.1 along reprocessing. This fact suggested that the chain scission was counteracted by the esterification reactions as was proposed in the FTIR analysis.

Melt flow index (MFI) of polymers is a critical parameter in material processing, and it is closely related to polymer molar mass. Table 1 shows a significant increase in MFI with increasing PLA reprocessing cycles. It was evidenced that reprocessing increased the chain mobility and fluency as had been previously reported for other polymers, such as polyamides [32], polyethylene, and polypropylene [33,34]. This fact can be related to the progressive decrease in molar mass attributed to the polymer chain cleavage and, therefore, degradation and poor crosslinking ability [35].

Optical properties of food packaging are critical for consumer perception and acceptance. Table 2 shows that PLA0 is luminous with green and yellow tones.

It was observed that reprocessing PLA systematically reduced the L* and b* values, and increased the a* value, which could be associated with the formation of chromophore compounds by the polymer chain scission [32]. Although reprocessed materials maintained the original tones (green and yellow), they were darker in comparison to those of rPLA, as was reported by [35]. These shifts are shown in color difference (ΔE) over five points, which means the observer would notice different colors between PLA0 and reprocessed materials.

### 3.2. Thermal Properties

**Figure 3:**
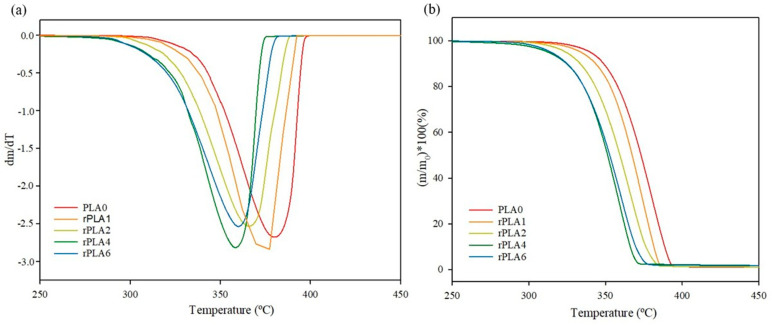
(**a**) TGA thermograms and (**b**) DTGA thermograms of reprocessed PLA samples.

TGA thermograms of reprocessed-PLA-based bottle preforms (Figure 3a) exhibited a single step of weight loss between 280 °C and 400 °C for all samples, which corresponded to the polymer decomposition [36]. Table 3 shows that both the onset or initial decomposition temperature (T_onset_) and the temperatures at maximum degradation rate (T_deg_) exhibited a slight but insignificant decrease after the first reprocessing, which became more noticeable after the fourth reprocessing, detecting a decrease in the onset mass loss temperature (T_onset_) of approx. 20 °C. The advance of the reprocessing caused a progressive reduction in the molar mass as shown in Table 1. Thus, shorter chains could be transformed into volatile products earlier, resulting in mass loss indicating decomposition at lower temperatures along with the reprocessing cycles.

**Figure 4:**
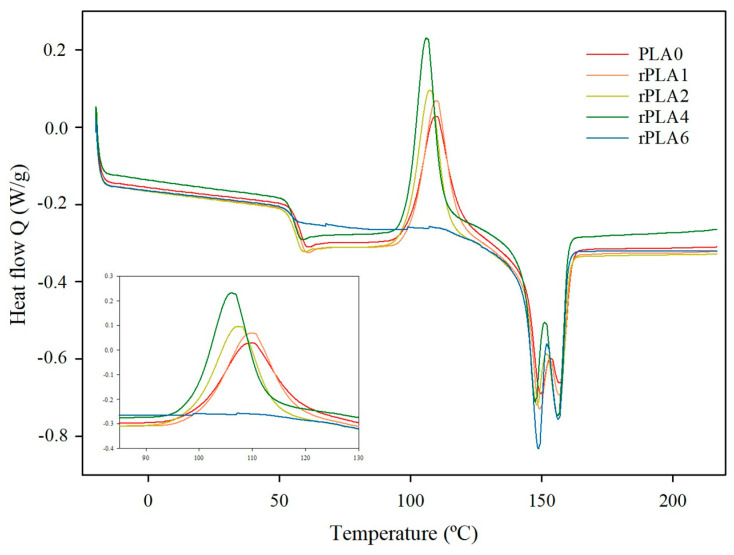
DSC thermograms of reprocessed rPLA samples.

Regarding DSC analysis, a progressive increase in the melting temperature and enthalpy, T_m1_, and *∆H_m_*, up to five cycles (Table 3), was observed, which indicated the formation of stronger crystallization cores as the PLA was reprocessed. Two types of PLA crystals, differing in order degree and thermal stability, were formed, associated with T_m1_ and T_m2_, respectively. These crystallization cores are associated with lower molar mass chains and small by-product molecules as reprocessing advances. Similar thermal behavior has been reported for accelerated aging and multi-extrusion in virgin PLA [37,38]. Nonetheless, as Figure 4 shows clearly, it is noted that after six reprocessing cycles, a severe increase in the crystallinity from 6.9 to 39.5% occurred, which indicated that, in this reprocessing level, the impact of the structural polymer changes and the molar mass is such that there was a greater formation of crystals during cooling because the chains were short enough to be easily reordered.

It is interesting to observe that from the sixth reprocessing cycle, rPLA6 had the ability to crystallize during cooling without cold crystallization as is shown in Figure 4. Conversely, T_g_ tended to diminish with reprocessing, but only had a statistically significant reduction at the sixth reprocessing cycle. Therefore, shorter polymer chains promoted fewer entanglements, and plasticizing and nucleating effects, obtaining a highly crystalline material, as was evidenced by the significantly higher χ_c_ value (Table 3).

### 3.3. Barrier Properties

Table 4 presents the water vapor permeability (WVP) and oxygen permeability (OP) values obtained for rPLA1 and rPLA2. For samples subjected to a higher number of reprocessing cycles (rPLA3–rPLA6), the preparation of oriented films by cast extrusion was not feasible due to their significantly increased melt flow, which prevented the production of films suitable for permeability testing.

The great increase in fluidity of the molten material made the coupling stage with the accessory to obtain the extruded cast films pleasantly difficult. The values of permeability for reprocessed samples were similar with those reported for pure extruded PLA where WVP and OP were in the order of 10^−14^ kg m m^−2^ s^−1^ Pa ^−1^ and 10^−7^ cm^3^ mm^−2^ day^−1^ Pa^−1^, respectively [39,40,41]. In addition to the reprocessing, the influence of relative humidity (RH) on the barrier properties was evaluated through a multivariance analysis (α = 0.05). Thus, WVP was influenced by RH (*p*-value = 0.0033) and reprocessing (*p*-value = 0.0188), but no factor interaction was found (*p*-value = 0.8166). In contrast, a significant interaction (*p*-value = 0.0011) between reprocessing and RH factors was found for OP, and each factor also had a significant influence. 

Although a decrease in permeability was expected since crystallinity increased from rPLA1 to rPLA2, the increase in WVP and OP by reprocessing is explained by the degradation and further polymer chain scission observed in the MFI and GPC results. The generation of hydrophilic polymeric chain ends of PLA (hydroxyl and carboxyl) enhanced the affinity with water vapor and oxygen molecules which can enhance their diffusion. This effect is especially favored for O_2_ at high RH. However, the change in barrier properties is not remarkable for WVP at RH50 and 90% and in the case of OP at RH50% since they remained in the same order (10^−15^ and 10^−7^, respectively). This slight increase in permeability has been previously reported for PLA materials subjected to accelerating aging processes [15,37] and multiple extrusions [42]. This fact was attributed to the shortening of polymer chains that were able to be rearranged and, therefore, decreased the free volume in the matrix [38].

Considering the barrier properties, reprocessed PLA could not be suitable for obtaining water bottles since oxidation of bottled water could result in the development of off-flavors. However, rPLA from water bottle preforms is appropriate to produce packaging materials for non-oxidative-sensitive food exposed to low RH, such as jams.

### 3.4. Biodegradation Results

**Figure 5:**
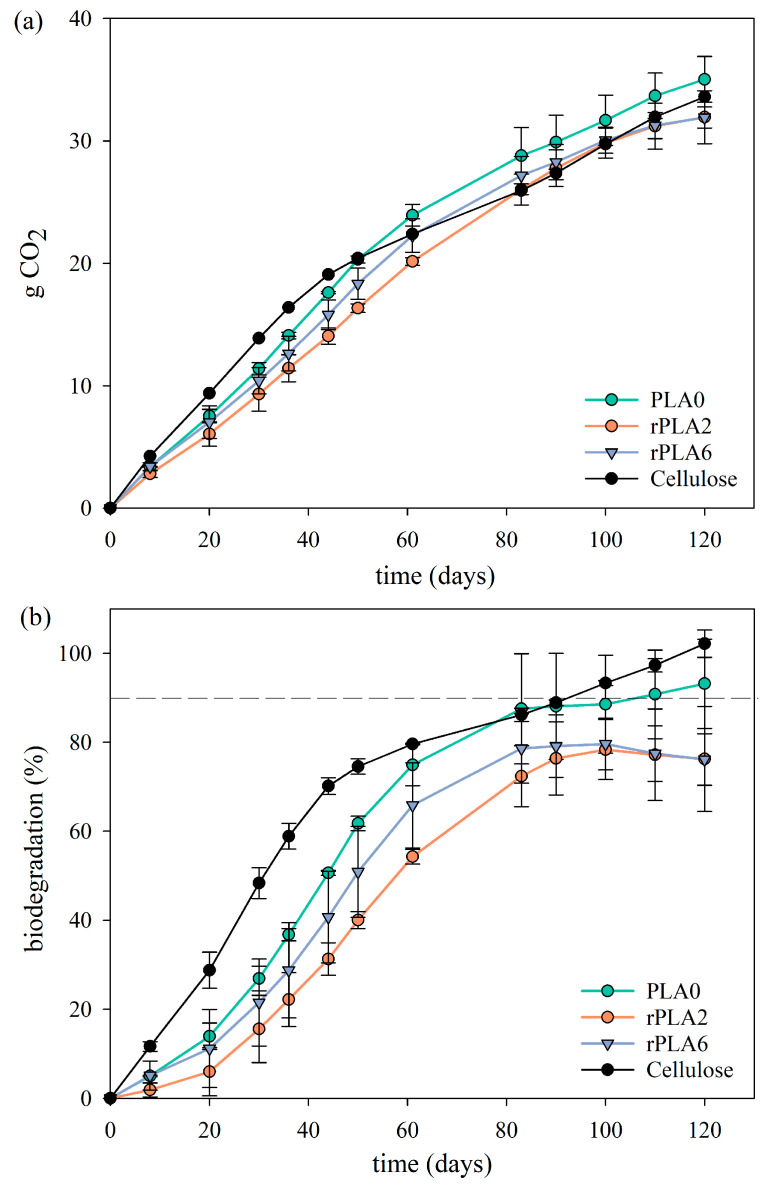
(**a**) CO_2_ evolution of multiple reprocessed-PLA-based water bottles; (**b**) biodegradation (%) kinetics.

Figure 5 shows the results of the biodegradation study under aerobic conditions. The parameters of the studied samples, including Nt and COT results, are included in Table S1. As Figure 5a shows, the CO_2_ production remained consistent for all samples throughout the 120-day testing period. All samples exhibited continuous behavior, with no staged biodegradation. Figure 5b shows the biodegradation kinetics of the samples, including a dashed line indicating 90% biodegradation, which is the threshold required to be achieved within 6 months under industrial composting conditions for a material to be classified as compostable. The curves revealed some interesting trends among the tested materials. First, it was observed that the cellulose control sample, followed by the original PLA bottle preform, exhibited the fastest biodegradation rates, both reaching approximately 90% biodegradation around day 110 of the experiment. It was also noteworthy that the biodegradation curves of all the samples clearly displayed a two-step profile: an initial, faster phase that accelerated around day 20 and extended until approximately day 60, followed by a deceleration of the process. This behavior has been previously shown in other works [43]. When comparing both reprocessed samples, although the rPLA6 sample exhibited higher crystallinity, its biodegradation proceeded more rapidly. Other works have stated crystallinity significantly affects biodegradation. At higher crystallinity degrees, the biodegradation takes more time because the chains in the crystalline regions are highly ordered, making them more compact and less mobile, which makes it more difficult for biodegrading agents to access the polymeric chains. Nevertheless, rPLA6 biodegraded slightly faster than rPLA2 due to its lower molar mass (Table 1). Lower molar mass increased the number of terminal groups and favored the autocatalytic hydrolysis of PLA, which partially offset the barrier effect of crystallinity. Kim et al. investigated how molecular weight and crystallinity affected the biodegradation rate of PLA. The authors reported that biodegradation rates decreased as molecular weight increased. At the same time, studies of PLAs with different crystallinities confirmed that lower crystallinity resulted in a faster biodegradation rate [44]. Although reprocessing significantly reduced the molar mass, crystallinity appeared to be the main factor influencing biodegradation, although molecular weight also affects the observed behavior.

## 4. Conclusions

The structural, optical, thermal, and barrier properties of reprocessed commercial PLA-based bottle preforms were evaluated. As expected, polymer chain cleavage by reprocessing cycles was evidenced by the continuous decrease in molar mass and increase in MFI values of reprocessed samples; however, the polydispersity index was maintained. The formation of new groups or compounds by polymer chain scission promoted a notable color difference (ΔE > 5) between the reprocessed samples. After four reprocessing cycles, the polymer chain scission promoted slight changes in the thermal properties; however, the sixth reprocessing of PLA resulted in a highly crystalline material. Nevertheless, this characteristic was not related to the barrier properties, since higher water and oxygen permeability were found for reprocessed samples, which were affected by a high-relative-humidity environment. These results suggest that PLA-based bottle preforms could be reprocessed for food packaging purposes other than bottles, where low humidity and thermal conditions are needed. Biodegradability tests revealed biodegradation kinetics and rates were highly dependent on both the molar mass and the crystallinity degree of a polymer. Future work including migration tests and mechanical property evaluations should be conducted in order to close more gaps in some important respects to gain a deeper understanding of these reprocessed plastics and their suitability for applications such as food packaging or other products.

## Figures and Tables

**Table 1:** Melt flow index (MFI), molar mass, and dispersity (Ð) of reprocessed PLA samples.

Sample	MFI (g 10 min^−1^)	$\bar{M_{n}}$	$\bar{M_{w}}$	Ð
PLA0	10.27 ± 0.20 ^d^	88,998 ± 1782 ^a^	186,702 ± 2587 ^a^	2.10 ± 0.02 ^ab^
rPLA1	14.77 ± 0.30 ^c^	74,463 ± 1410 ^b^	155,217 ± 1178 ^b^	2.08 ± 0.04 ^a^
rPLA2	14.59 ± 0.09 ^c^	72,292 ± 1907 ^c^	152,676 ± 2094 ^c^	2.11 ± 0.03 ^c^
rPLA4	15.81 ± 0.09 ^b^	62,652 ± 1503 ^d^	132,388 ± 1960 ^d^	2.11 ± 0.02 ^d^
rPLA6	15.30 ± 0.37 ^a^	53,972 ± 453 ^e^	113,942 ± 1355 ^e^	2.11 ± 0.01 ^bc^

Average values with different letters (a–e) within a column show significant differences (*p* < 0.05) between the samples according to ANOVA analysis.

**Table 2:** CIELab coordinates of reprocessed PLA.

Sample		L*	a*	b*	ΔE*
PLA0	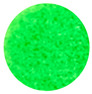	71.1 ± 0.1 ^a^	−34.8 ± 0.1 ^e^	34.4 ± 0.1 ^a^	-
rPLA1	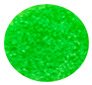	66.7 ± 0.2 ^b^	−31.7 ± 0.2 ^d^	32.4 ± 0.3 ^b^	5.8 ± 0.4 ^d^
rPLA2	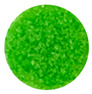	63.6 ± 0.1 ^c^	−24.5 ± 0.4 ^c^	30.4 ± 1.0 ^c^	13.4 ± 0.7 ^c^
rPLA4	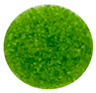	60.6 ± 0.3 ^d^	−16.9 ± 0.1 ^b^	27.2 ± 0.2 ^d^	22.1 ± 0.1 ^b^
rPLA6	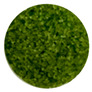	47.3 ± 0.1 ^e^	−9.30 ± 0.1 ^a^	25.7 ± 0.6 ^e^	35.9 ± 0.1 ^a^

Average values with different letters (a–e) within a column show statistically significant differences (*p* < 0.05) between the samples.

**Table 3:** Thermal parameters of reprocessed PLA.

Sample	T_g_ (°C)	T_cc_ (°C)	T_m1_ (°C)	T_m2_ (°C)	ΔH_cc_ (J/g)	ΔH_m_ (J/g)	χ_c_ (%)	T_onset_ (°C)	T_deg_ (°C)
PLA0	57.9 ± 0.4 ^a^	108.4 ± 2.0 ^ab^	149.0 ± 0.7 ^b^	156.8 ± 0.8 ^d^	26.4 ± 0.7 ^a^	31.6 ± 0.4 ^c^	5.6 ± 1.1 ^b^	356.6 ± 2.3 ^c^	376.6 ± 4.3 ^b^
rPLA1	57.9 ± 0.3 ^a^	110.0 ± 0.3 ^a^	149.1 ± 0.1 ^b^	156.6 ± 0.3 ^c^	28.1 ± 0.6 ^a^	31.7± 0.5 ^c^	3.8 ± 1.2 ^b^	350.6 ± 1.9 ^bc^	374.2 ± 2.9 ^b^
rPLA2	56.7 ± 0.2 ^a^	107.3 ± 0.2 ^ab^	156.3 ± 0.1 ^a^	156.3 ± 0.1 ^b^	25.7 ± 1.4 ^a^	33.4 ± 0.1 ^b^	8.2 ± 1.4 ^b^	342.6 ± 6.0 ^ab^	369.1 ± 6.0 ^ab^
rPLA4	56.5 ± 0.4 ^a^	106.2 ± 0.2 ^b^	156.0 ± 0.1 ^a^	156.0 ± 0.2 ^a^	28.4 ± 2.1 ^a^	34.8 ± 1.1 ^b^	6.9 ± 3.5 ^b^	337.3 ± 4.6 ^a^	357.8 ± 8.7 ^a^
rPLA6	54.8 ± 1.0 ^b^	-	148.6 ± 0.3 ^b^	156.2 ± 0.3 ^b^	-	36.7 ± 0.7 ^a^	39.5 ± 0.7 ^a^	337.9 ± 6.4 ^a^	359.7 ± 9.4 ^a^

Mean values with different letters (a–d) within a column show significant differences (*p* < 0.05) between the samples.

**Table 4:** Barrier properties of reprocessed rPLA samples.

Sample	WVP (kg m m^−2^ s^−1^ Pa ^−1^)·10^−15^		OP (cc m m^−2^ day^−1^ Pa^−1^)·10^−7^	
	RH 50%	RH 90%	RH 50%	RH 90%
rPLA1	4.1 ± 0.9	5.7 ± 0.6	2.1 ± 0.4	24.8 ± 4.7
rPLA2	5.3 ± 0.6	6.8 ± 0.3	5.3 ± 0.9	225.7 ± 33.1

## Data Availability

The data that support the findings of this study are available from the corresponding author upon request due to privacy reasons.

## Supplementary Materials

The following supporting information can be downloaded at: https://www.mdpi.com/article/10.3390/polym17182542/s1, SM-1. Plastic transformation processes of rPLA samples; SM-2. Molar mass of reprocessed rPLA samples; SM-3. Characterization of samples for biodegradation tests.

## References

[1] da Silva Pens C.J., Klug T.V., Stoll L., Izidoro F., Flores S.H., de Oliveira Rios A. (2024). Poly (Lactic Acid) and Its Improved Properties by Some Modifications for Food Packaging Applications: A Review. Food Packag. Shelf Life.

[2] Huang S., Dong Q., Che S., Li R., Tang K.H.D. (2025). Bioplastics and Biodegradable Plastics: A Review of Recent Advances, Feasibility and Cleaner Production. Sci. Total Environ..

[3] Agüero A., Morcillo M.d.C., Quiles-Carrillo L., Balart R., Boronat T., Lascano D., Torres-Giner S., Fenollar O. (2019). Study of the Influence of the Reprocessing Cycles on the Final Properties of Polylactide Pieces Obtained by Injection Molding. Polymers.

[4] Majgaonkar P., Hanich R., Malz F., Brüll R. (2021). Chemical Recycling of Post-Consumer PLA Waste for Sustainable Production of Ethyl Lactate. Chem. Eng. J..

[5] Dedieu I., Peyron S., Gontard N., Aouf C. (2022). The Thermo-Mechanical Recyclability Potential of Biodegradable Biopolyesters: Perspectives and Limits for Food Packaging Application. Polym. Test..

[6] Terzopoulou Z., Bikiaris D.N. (2024). Biobased Plastics for the Transition to a Circular Economy. Mater. Lett..

[7] Cosate de Andrade M.F., Souza P.M.S., Cavalett O., Morales A.R. (2016). Life Cycle Assessment of Poly(Lactic Acid) (PLA): Comparison Between Chemical Recycling, Mechanical Recycling and Composting. J. Polym. Environ..

[8] Rebolledo-Leiva R., Ladakis D., Ioannidou S.-M., Koutinas A., Moreira M.T., González-García S. (2023). Attributional and Consequential Life Cycle Perspectives of Second-Generation Polylactic Acid: The Benefits of Integrating a Recycling Strategy. J. Clean. Prod..

[9] Maga D., Hiebel M., Thonemann N. (2019). Life Cycle Assessment of Recycling Options for Polylactic Acid. Resour. Conserv. Recycl..

[10] Fredi G., Dorigato A. (2021). Recycling of Bioplastic Waste: A Review. Adv. Ind. Eng. Polym. Res..

[11] Sun C., Wei S., Tan H., Huang Y., Zhang Y. (2022). Progress in Upcycling Polylactic Acid Waste as an Alternative Carbon Source: A Review. Chem. Eng. J..

[12] Ramos-Hernández T., Robledo-Ortíz J.R., González-López M.E., del Campo A.S.M., González-Núñez R., Rodrigue D., Pérez Fonseca A.A. (2023). Mechanical Recycling of PLA: Effect of Weathering, Extrusion Cycles, and Chain Extender. J. Appl. Polym. Sci..

[13] Shojaeiarani J., Bajwa D.S., Rehovsky C., Bajwa S.G., Vahidi G. (2019). Deterioration in the Physico-Mechanical and Thermal Properties of Biopolymers Due to Reprocessing. Polymers.

[14] Finnerty J., Rowe S., Howard T., Connolly S., Doran C., Devine D.M., Gately N.M., Chyzna V., Portela A., Bezerra G.S.N. (2023). Effect of Mechanical Recycling on the Mechanical Properties of PLA-Based Natural Fiber-Reinforced Composites. J. Compos. Sci..

[15] Beltrán F.R., Gaspar G., Dadras Chomachayi M., Jalali-Arani A., Lozano-Pérez A.A., Cenis J.L., de la Orden M.U., Pérez E., Martínez Urreaga J.M. (2021). Influence of Addition of Organic Fillers on the Properties of Mechanically Recycled PLA. Environ. Sci. Pollut. Res..

[16] Velghe I., Buffel B., Vandeginste V., Thielemans W., Desplentere F. (2023). Review on the Degradation of Poly(Lactic Acid) during Melt Processing. Polymers.

[17] Wang W., Ye G., Fan D., Lu Y., Shi P., Wang X., Bateer B. (2021). Photo-Oxidative Resistance and Adjustable Degradation of Poly-Lactic Acid (PLA) Obtained by Biomass Addition and Interfacial Construction. Polym. Degrad. Stab..

[18] (2022). Plastics—Determination of the Melt Mass-Flow Rate (MFR) and Melt Volume-Flow Rate (MVR) of Thermoplastics—Part 1: Standard Method.

[19] Navneet, Martinez M.M., Joye I.J. (2024). Heat-Treated Bean Flour: Exploring Techno-Functionality via Starch-Protein Structure-Function Analysis. Food. Hydrocoll..

[20] Romani A., Perusin L., Ciurnelli M., Levi M. (2024). Characterization of PLA Feedstock after Multiple Recycling Processes for Large-Format Material Extrusion Additive Manufacturing. Mater. Today Sustain..

[21] (2017). Sheet Materials—Determination of Water Vapour Transmission Rate (WVTR)—Gravimetric (Dish) Method.

[22] (2018). Determination of the Ultimate aerobic Biodegradability of Plastic Materials Under Controlled Composting Conditions—Method by Analysis of Evolved Carbon Dioxide. Part 2: Gravimetric Measurement of Carbon Dioxide Evolved in a Laboratory-Scale Test.

[23] Palsikowski P.A., Kuchnier C.N., Pinheiro I.F., Morales A.R. (2018). Biodegradation in Soil of PLA/PBAT Blends Compatibilized with Chain Extender. J. Polym. Environ..

[24] Oh J., Park S.B., Cha C., Hwang D.K., Park S.A., Park J., Oh D.X., Jeon H., Koo J.M. (2024). Structural Evaluation of Poly(Lactic Acid) Degradation at Standardized Composting Temperature of 58 Degrees. Chemosphere.

[25] Siddiqui M.N., Redhwi H.H., Tsagkalias I., Vouvoudi E.C., Achilias D.S. (2021). Development of Bio-Composites with Enhanced Antioxidant Activity Based on Poly(Lactic Acid) with Thymol, Carvacrol, Limonene, or Cinnamaldehyde for Active Food Packaging. Polymers.

[26] Salah L.S., Ouslimani N., Danlée Y., Beltrán F.R., Huynen I., de la Orden M.U. (2023). Investigation of Mechanical Recycling Effect on Electromagnetic Properties of Polylactic Acid (PLA)–Nanoclay Nanocomposites: Towards a Valorization of Recycled PLA Nanocomposites. Compos. Part C Open Access.

[27] Garcia Gonçalves L.M., Rocha Rigolin T., Maia Frenhe B., Prado Bettini S.H. (2020). On the Recycling of a Biodegradable Polymer: Multiple Extrusion of Poly (Lactic Acid). Mater. Res..

[28] Velásquez E., Guerrero Correa M., Garrido L., Guarda A., Galotto M.J., López de Dicastillo C., Parameswaranpillai J., Mavinkere Rangappa S., Gulihonnehalli Rajkumar A., Siengchin S. (2021). Food Packaging Plastics: Identification and Recycling. Recent Developments in Plastic Recycling.

[29] Beltrán F.R., Infante C., de la Orden M.U., Martínez Urreaga J. (2019). Mechanical Recycling of Poly(Lactic Acid): Evaluation of a Chain Extender and a Peroxide as Additives for Upgrading the Recycled Plastic. J. Clean. Prod..

[30] Lee D., Lee Y., Kim I., Hwang K., Kim N. (2022). Thermal and Mechanical Degradation of Recycled Polylactic Acid Filaments for Three-Dimensional Printing Applications. Polymers.

[31] Botta L., Scaffaro R., Sutera F., Mistretta M.C. (2017). Reprocessing of PLA/Graphene Nanoplatelets Nanocomposites. Polymers.

[32] Morales J., Rodrigue D. (2025). The Effect of Reprocessing and Moisture on Polyamide Recycling: A Focus on Neat, Composites, and Blends. Macromol. Mater. Eng..

[33] Verberckmoes A., Fernandez E., Martins C., Reyes P., Cardon L., D’hooge D.R., Edeleva M. (2024). Morphological Analysis of Mechanically Recycled Blends of High Density Polyethylene and Polypropylene with Strong Difference in Melt Flow Index. Polymer.

[34] Velásquez E., de Dicastillo C.L., Rojas A., Garrido L., Pérez C.J., Lira M., Guarda A., Galotto M.J. (2024). Multiple Mechanical Recycling of a Post-Industrial Flexible Polypropylene and Its Nanocomposite with Clay: Impact on Properties for Food Packaging Applications. Food Packag. Shelf Life.

[35] Plouzeau M., Belyamani I., Fatyeyeva K., Marais S., Kobzar Y., Cauret L. (2023). Recyclability of Poly(Hydroxybutyrate-Co-Hydroxyhexanoate) (PHBH) for Food Packaging Applications. Food Packag. Shelf Life.

[36] Martin-Perez L., Contreras C., Chiralt A., Gonzalez-Martinez C. (2025). Active Polylactic Acid (PLA) Films Incorporating Almond Peel Extracts for Food Preservation. Molecules.

[37] Beltrán F.R., Arrieta M.P., Gaspar G., de la Orden M.U., Urreaga J.M. (2020). Effect of Lignocellulosic Nanoparticles Extracted from Yerba Mate (Ilex Paraguariensis) on the Structural, Thermal, Optical and Barrier Properties of Mechanically Recycled Poly(Lactic Acid). Polymers.

[38] Zenkiewicz M., Richert J., Rytlewski P., Moraczewski K., Stepczyńska M., Karasiewicz T. (2009). Characterisation of Multi-Extruded Poly(Lactic Acid). Polym Test..

[39] Muñoz-Shugulí C., Rodríguez-Mercado F., Guarda A., Galotto M.J., Jiménez A., Garrigós M.C., Ramos M. (2024). Release and Disintegration Properties of Poly(Lactic Acid) Films with Allyl Isothiocyanate-β-Cyclodextrin Inclusion Complexes for Active Food Packaging. Molecules.

[40] Fan Z., Fu L., Lan L., Dan Y., Jiang L., Huang Y. (2025). Effect of Poly(Vinyl Alcohol)-g-Poly(Lactic Acid) on the Oxygen Barrier Performance of Poly(Lactic Acid)-Based Film. Int. J. Biol. Macromol..

[41] Marano S., Laudadio E., Minnelli C., Stipa P. (2022). Tailoring the Barrier Properties of PLA: A State-of-the-Art Review for Food Packaging Applications. Polymers.

[42] Beltrán F.R., Lorenzo V., Acosta J., de la Orden M.U., Martínez Urreaga J. (2018). Effect of Simulated Mechanical Recycling Processes on the Structure and Properties of Poly(Lactic Acid). J. Environ. Manag..

[43] D’Amario J., Limsukon W., Bher A., Auras R. (2025). Impact of Hydrolysis Pretreatment on the Compostability of Biodegradable Poly(Caprolactone) and Poly(Lactic Acid) Films. RSC Appl. Polym..

[44] Kim M.N., Park S.T. (2010). Degradation of Poly(L-Lactide) by a Mesophilic Bacterium. J. Appl. Polym. Sci..

