# Supplementary material for: Effect of Degradation During Multiple Primary Mechanical Recycling Processes on the Physical Properties and Biodegradation of Commercial PLA-Based Water Bottles
Source: Polymers (Basel). 2025 Sep 20;17(18):2542. doi: 10.3390/polym17182542 (PMC12473880; doi:10.3390/polym17182542)
Supplement: Supplementary file 1 [file polymers-17-02542-s001.zip › polymers-3872010-supplementary.pdf]

Supplementary material

Effect of degradation during multiple primary mechanical recycling processes on the physical properties and biodegradation of commercial PLA-based water bottles

C. Muñoz-Shugulí[1, 2], D. Morán[3], E. Velásquez[4], J.M. López Vilariño[5], C. López-de-Dicastillo[3]*

[1] Faculty of Sciences, Escuela Superior Politécnica de Chimborazo (ESPOCH), EC060106, Riobamba, Ecuador.

[2] Group for Research and Innovation in Food Packaging, Riobamba, EC060107, Ecuador.

[3] Packaging Group, Institute of Agrochemistry and Food Technology (IATA-CSIC), Av. Agustín Escardino 7, 46980 Paterna, Valencia, Spain.

[4] Packaging Innovation Center (LABEN-Chile), University of Santiago of Chile (USACH), Santiago 9170201, Chile

[5] Hijos de Rivera SAU, José María Rivera Corral 6, A Coruña, Spain

SM-1. Plastic transformation processes of rPLA samples

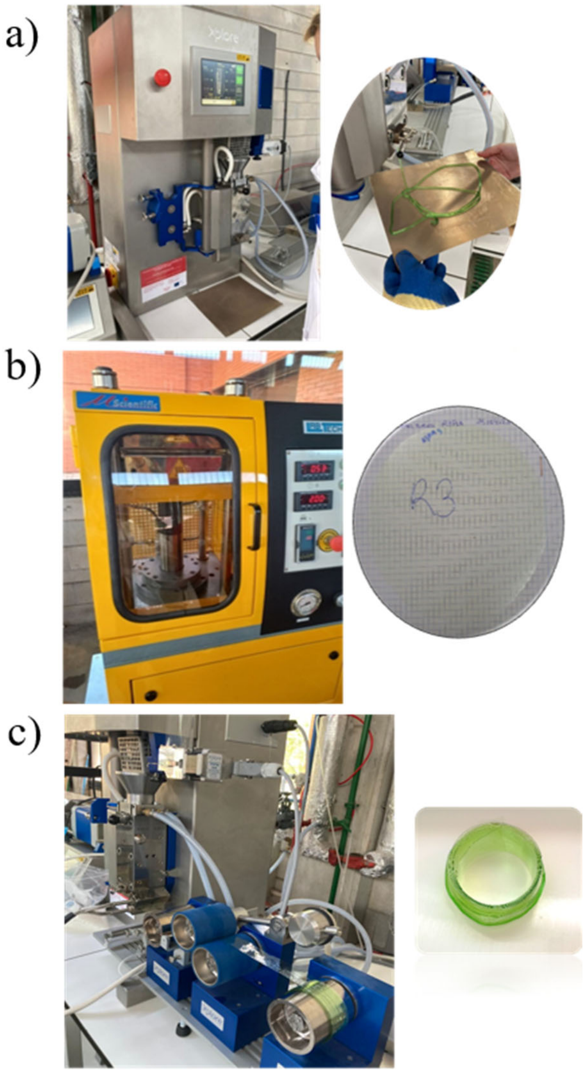

Figure S1 a) Co-rotating twin-screw mini extruder; b) Hot plate press; and c) Cast-extrusion of reprocessed rPLA samples

SM-2. Molar mass results of reprocessed rPLA samples

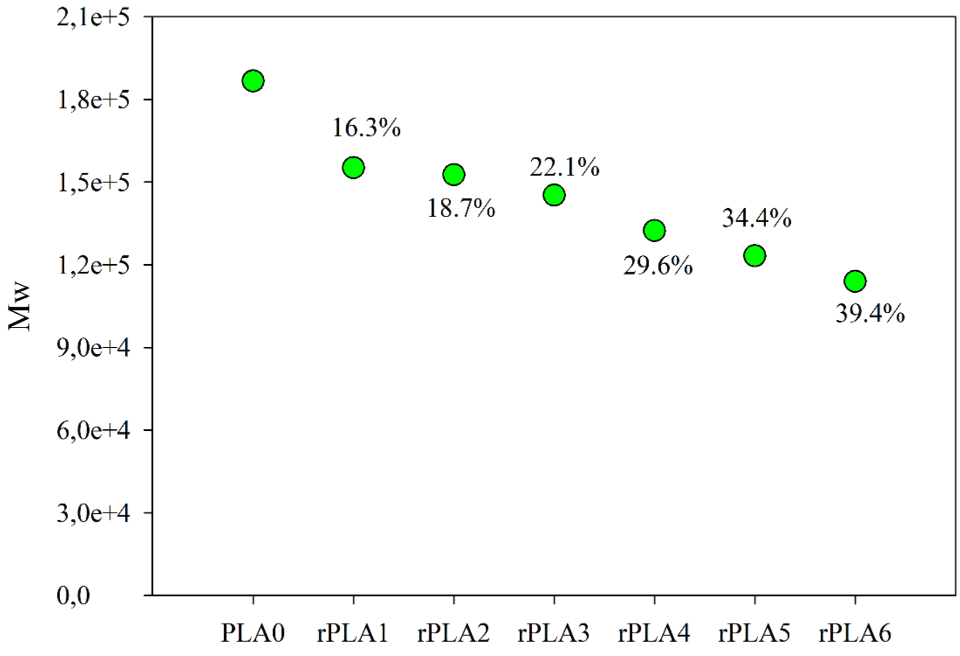

Figure S2 Molecular weight (Mw) of reprocessed PLA. In numbers percentage of reduction respect the initial molecular weight

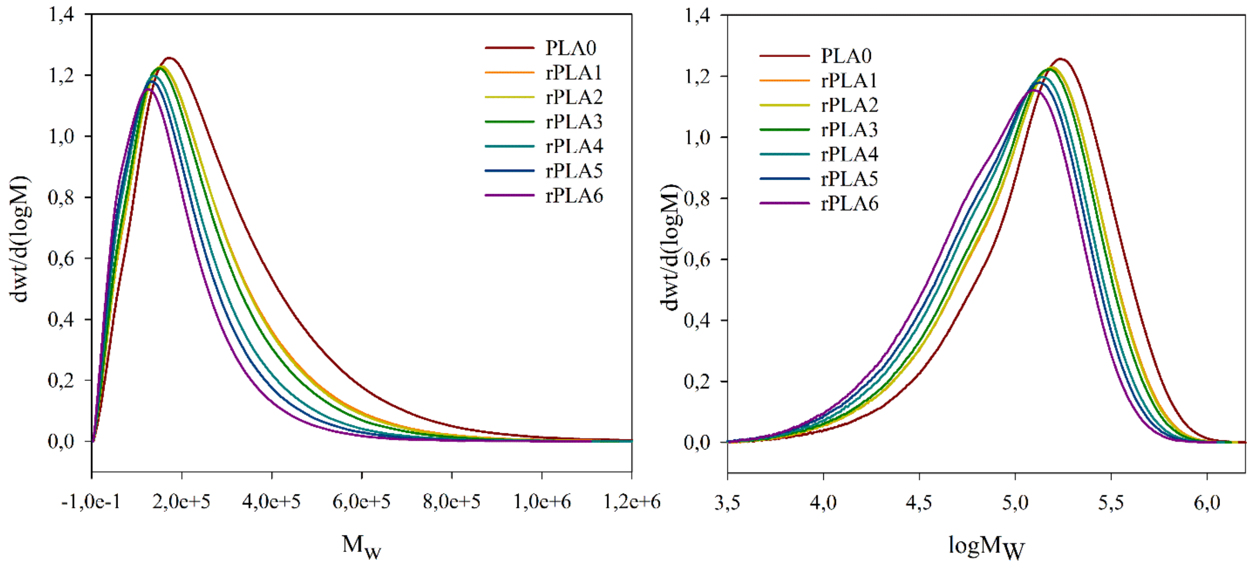

Figure S3 Molecular weight (Mw) curves of reprocessed PLA samples.

SM-3. Characterization of samples for biodegradation tests

Samples were also analyzed through two supporting assays:

i) Determination of Total Nitrogen (Nt):

- Plastic matrix: Internal procedures B-N211 and B-N214 according to ASTM D5291.

ii) Determination of Total Organic Carbon (TOC)

- Plastic matrix: Internal procedures B-N211 and B-N214 according to ASTM D5291.

The parameters of the test samples are shown in the following Table 1S.

Table S1

Parameters of analyzed samples.

Sample	*Cellulose*	BOT_0	*RBOT*	$RBOT_{+1}$
Humidity (%)	insignificant	insignificant	insignificant	insignificant
Ntotal (%sms)	0.25	0.10	0.10	0.10
Volatile solids (% sms)	100	ND	ND	ND
Appearance	white powder	grinded	grinded	grinded
Geometry of particles	< 20 µm	< 3 mm	< 3 mm	< 3 mm
Additives	No	No	No	No
COT (%sms)	41.70	50.10	50.20	50.10
dry mass (g)	~ 10.05	~ 10.02	~ 10.03	~ 10.03
COT (g)	~ 4.21	~ 5.02	~ 5.04	~ 5.02
$ThCO_2$ (g)	~ 15.43	~ 18.40	~ 18.46	~ 18.42
